# Physical‐activity support for people with intellectual disabilities: development of a tool to measure behavioural determinants in direct support professionals

**DOI:** 10.1111/jir.12631

**Published:** 2019-05-20

**Authors:** L. W. M. Bossink, A. A. J. van der Putten, H. A. Steenbergen, C. Vlaskamp

**Affiliations:** ^1^ Department of Special Needs Education and Youth Care University of Groningen Groningen The Netherlands; ^2^ Applied Sciences in Health Care and Nursing Hanze University of Applied Sciences Groningen Groningen The Netherlands; ^3^ Department of Health Psychology University of Groningen, University Medical Center Groningen Groningen The Netherlands

**Keywords:** behavioural determinants, direct support professionals, implementation, item response theory, people with intellectual disabilities, physical activity

## Abstract

**Background:**

Physical‐activity approaches for people with intellectual disabilities (ID) are more likely to be effective and sustainable if they also target direct support professionals' behaviour. However, no tools to measure the behavioural determinants for direct support professionals are available as of yet. This study aims to construct a self‐report tool to measure direct support professionals' behavioural determinants in physical‐activity support for people with ID and to analyse its psychometric properties.

**Methods:**

The tools' sub‐scales and items corresponded with a proposed conceptual model. A pilot study was carried out to investigate and improve content validity. Construct validity and measurement precision were examined using item response theory models with data from a convenience sample of 247 direct support professionals in the support of people with ID.

**Results:**

Results supported the three theory‐driven behaviour scales and indicated reasonable to good construct validity. The marginal reliability for the scales ranged from 0.84 to 0.87, and adequate measurement precision along the latent continua was found.

**Conclusions:**

The tool appears to be promising for measuring the behavioural determinants of direct support professionals for the physical‐activity support of people with ID and has potential as a tool for identifying areas to focus on for interventions and policies in the future.

## Background

There is growing recognition that interventions aimed at promoting the participation in physical activity of people with intellectual disabilities (ID) should also target the physical and social environment of these people (Peterson *et al*. 2008; Heller *et al*. 2011; Bergström *et al*. 2013; Kuijken *et al*. 2016; Bossink *et al*. 2017; Steenbergen *et al*. 2017). A large and essential part of this physical and social environment can be attributed to the quality and content of the support provided by direct support professionals (Buntinx & Schalock 2010). The content of the support received from direct support professionals has turned out to predict the physical‐activity participation in adults with mild to moderate ID (Peterson *et al*. 2008). Moreover, support from others, such as direct support professionals, is often indicated as being an important factor that influences whether people with mild to moderate ID participate in physical activity (Kuijken *et al*. 2016; Bossink *et al*. 2017). Although these findings were biased towards the support of people with mild to moderate ID, it is known that engaging people with a combination of profound intellectual and severe motor disabilities in physical activities requires intensive effort and support from others (Nakken & Vlaskamp 2007; Van der Putten *et al*. 2017).

Targeting and influencing the support of direct support professionals, however, requires a thorough understanding of their perspective. Recently, a theory‐informed qualitative study explored the perspective of direct support professionals as regards physical‐activity support for people with ID (Bossink *et al*. 2019). Underpinned by valid theoretical frameworks for behaviour and behavioural change (Michie *et al*. 2011; Cane *et al*. 2012), various influences on the behaviour of direct support professionals were explored as related to the three essential sources of the nature of behaviour (e.g. capability, opportunity and motivation). A conceptual model was proposed comprising the influential factors that facilitate or impede physical‐activity support related to the capability, to the opportunities afforded and, subsequently, to the motivation of direct support professionals in terms of engaging in physical‐activity support (Bossink *et al*. 2019). Another important finding included in this conceptual model concerns those characteristics of people with ID that affect direct support professional behaviour vis‐à‐vis physical‐activity support.

Because the perspectives presented in the qualitative research findings were wide ranging (Bossink *et al*. 2019), an additional step is needed to accurately measure the differences in direct support professional behaviour in order to promote physical‐activity participation in people with ID. To our knowledge, no validated tools exist to measure the behavioural determinants of direct support professionals in the context of the physical‐activity support for people with ID. This study will therefore attempt to develop a validated tool based on the theoretical knowledge of behaviour and behaviour changes in direct support professionals regarding physical‐activity support for people with ID. This study's main focus is on the initial evaluation of the tool's psychometric properties. This tool can subsequently be used to investigate direct support professional behaviour regarding their support in promoting physical activity and to identify areas for future interventions and policies.

## Methods

### Study design and participant selection

A cross‐sectional approach was used. The inclusion criteria for the participants were as follows: (1) professional supporting a group of people with ID in a living unit and/or activity centre and (2) being directly in contact with people with ID for most of the working time. No reward or incentive was offered for participation. The participants were mainly recruited from 10 residential facilities in the Netherlands. Each facility was allowed to decide how to internally distribute the invitation for participation in this study. An indication of the overall response rate was given by calculating the response rate for the four participating facilities that invited professionals to participate by e‐mail (21.4% response rate). Awareness for this study was raised by online advertising in the other six facilities. In addition, participants were also recruited via a national information platform for direct support professionals and by social media.

In total, 395 potential participants visited the online application that introduced the tool (260 from the facilities and 135 from social media/national information platforms). Of these, 363 chose to participate and completed the screening questions (i.e. the inclusion criteria for this study). A total of 28 did not meet our inclusion criteria. A further 50 did meet our inclusion criteria but exited the questionnaire after the screening, and another 38 completed less than half of the items (<21 items).

A convenience sample of 247 participants was used in this study. Table 1 shows the characteristics of the participants.

**Table 1:** Participant characteristics (*n* = 213–216†)

Characteristic	*n* (%)†	
Gender		
Female	182 (84)	
Male	34 (16)	
Profession		
Direct support professional	93 (43)	
Senior direct support professional‡	123 (57)	
Educational level		
Basic vocational education	3 (1)	
Intermediate vocational education	130 (60)	
Higher professional education	78 (36)	
Master's degree	5 (2)	
Characteristic	Mean (SD)	Range
Age (years)	42.4 (11.6)	22–65
Years employed as direct support professional in the support of people with ID	16.6 (10.2)	0.5–46
Years employed at current organisation	13.8 (9.3)	0.5–44
Average working time per week (h)	26.1 (6.4)	6–40

† No category has the same total *n* value, as a different number of responses were missing for each question.

‡ Senior direct support professionals have additional tasks such as coordinating the planning of multidisciplinary meetings, contact with parents and partial responsibility for the content of individual support plans, etc.

ID, intellectual disability.

### Development of the capability, opportunity and motivation sub‐scales

The tools' sub‐scales and items correspond to a proposed conceptual model for understanding direct support professional behaviour in their physical‐activity support for people with ID (Bossink *et al*. 2019) and were supplemented with the results of a systematic review identifying barriers and facilitators of physical activity in people with ID (Bossink *et al*. 2017). The sub‐scale *Capability* represents the professionals' psychological and physical ability to enact a behaviour, which includes having the necessary knowledge and skills. *Opportunity* is defined as any circumstance in the physical or social environment that influences a behaviour: all factors that are external to the professional. *Motivation* represents all those brain processes that energise and direct the behaviour of the professional (Michie *et al*. 2011). These sources (i.e. the three sub‐scales) interact to generate the behaviour of interest (i.e. direct support professional behaviour regarding their support in promoting physical activity) (Michie *et al*. 2011). The influencing factors facilitating or impeding physical‐activity support known in the literature were, for this study, compiled into items that were presumed to be reflective indicators of the three different sources of direct support professional behaviour. Lower item scores reflect an influencing factor that acts as a barrier; higher scores indicate a facilitator. The item distribution among sub‐scales is based on the number of influences on the underlying construct known in the literature. The selection and designing process was discussed during regular meetings with the research group. Item‐writing guidelines were used (Mellenbergh 2011, pp. 73–78; Van Sonderen *et al*. 2013). In addition, a five‐point Likert scale (from 0 ‘disagree’ to 4 ‘agree’) was used for the different response categories of each item (Krosnick & Fabrigar 1997, as cited in Mellenbergh 2011, p. 78).

Two content experts were involved to improve content validity and to assess the applicability for current practice in the work of direct support professionals. One expert worked as a physiotherapist in a large‐scale residential facility, and the other worked as a movement scientist. Both experts have experience with developing questionnaires for research and professional purposes. After feedback from the expert panel, the first draft of the tool was developed comprising 41 items: 8 for the sub‐scale ‘capability’, 15 for the sub‐scale ‘opportunity’ and 18 for the sub‐scale ‘motivation’.

With this tool, a pilot study was carried out with a convenience sample of 10 direct support professionals, who were not enrolled in this study's sample. Each direct support professional was asked to complete the first draft of the tool, to fill out a demographic questionnaire and to finish a retrospective evaluation form – all online. The demographic questionnaire included questions about the characteristics of the people with whom they work (e.g. age, level of ID and additional impairments), their own characteristics (e.g. age, gender, profession and employment years) and characteristics of their organisations. The evaluation form included questions about the time needed to complete the tool, the clarity and completeness of the instructions at the start and in the course of completing the tool, the clarity and applicability of individual items and their response options in the tool and the completeness of the tool in terms of the physical‐activity support topic. The proposed tool, a demographic questionnaire, and an evaluation form were made available online using *Qualtrics* research software.

The pilot results were discussed with the research group and two field experts, which resulted in some adjustments. One item on education was removed from the sub‐scale ‘capability’ and translated into an organisational characteristic about whether or not they were trained in physical‐activity support and what sort of education they had received, which was then relocated in the demographic questionnaire. Another item on practical support was added to the tool and was attributed to the sub‐scale ‘opportunity’. Based on the pilot results, we also added ‘expected time costs’ to the introduction section and screening questions. Furthermore, we decided to add a question to the demographic questionnaire about the role of the physiotherapist in their organisation.

A final 41‐item self‐reported tool was proposed, with seven items covering the capability construct, 16 the opportunity construct and 18 the motivational construct. *Qualtrics* research software was again used to make both the adapted demographic questionnaire and the proposed tool available online. The psychometric properties of the tool were examined in this study.

### Statistical analyses

The descriptive statistics were computed first. Raw item scores were described according to mean (standard deviation), and the frequency scores of the response options were given. Response categories were collapsed for further analyses, in case too few participants had chosen a response option (minimum of 12 ratings for a response option).

The psychometric properties were analysed using an item response theory (IRT) model separately for the three sub‐scales proposed. IRT is a statistical theory consisting of mathematical models describing the relationships between the properties of single items of a tool, the underlying construct that a tool proposes to measure and respondents' answers to any item (Kline 2005). Compared with classical test theory, IRT models generate much richer item level information and greater detail on the tool's reliability (Nguyen *et al*. 2014). Based on the underlying theory, unidimensionality for the three sub‐scales was warranted. The different sub‐scales were then calibrated under a polytomous item response model using the R mirt package version 1.27.1 (Chalmers *et al*. 2018) in the open‐source software environment R version 3.4.3 (R Development Core Team 2017). The marginal maximum likelihood estimation was used to estimate item parameters (Bock & Aitkin 1981). Samejima's (1969) graded response models were estimated, which are potentially useful models when item response options lie on an ordered but categorical level. Samejima's model is a polytomous extension of the two‐parameter logistic model for dichotomous item responses and was chosen over the more restricted model of Muraki (1990), because this model allows for item response options that do not have to be the same across items (Kline 2005, pp. 131–137).

For Samejima's model, the item characteristic curve that relates the probability of an item response to the underlying construct (denoted *θ*), measured by the item set, is characterised by two parameters: a slope parameter (denoted *α*) and the thresholds category parameters (denoted as *β*). *α* describes how well an item can differentiate along *θ* and, similar to factor loadings, how well the item relates to the construct measured. A reasonable range for *α* is from 0.5 to 3.0 (Baker, as cited in Toland 2014). *β* defines the point on *θ* at which 50% of the respondents would choose the designated response category or higher. Every respondent has a 100% probability of choosing the lowest category or higher, so there are (number of response categories – 1) *β*'s for each item (Kline 2005, pp. 131–132). *β* generally ranges from −2 to 2, but it is not uncommon for this parameter to range between −3 and 3 (Toland 2014).

The information functions (Toland 2014) are the IRT equivalent of reliability. Each item has its own item information function (IIF) shaped by its item parameters. With IIFs, the amount of precision for each item was gathered for a particular location or across a range on *θ* (Toland 2014). In addition, it was used to see how much information an item is adding to the entire scale and where that information is occurring along *θ* (Toland 2014). For each scale, IIFs were combined into a test information function illustrating the precision of this scale for each score level of *θ*. Moreover, marginal reliability was estimated representing a value that summarised the precision for the entire range of a scale (similar to traditional reliability; Green *et al*. 1984). Finally – and in addition – the IRT score estimates (*θ* for each respondent on the scale) and their standard errors were assessed.

## Results

### Item distributions

Table 2 presents the average items scores and frequency scores of the response options for items within the different sub‐scales. The participants, for the most part, agreed or partly agreed with the items in the capability scale, especially on the items covering their awareness, knowledge and skills (mean score > 3.0). Within the opportunity scale, the response options *partly agree* and *agree* were, on average, slightly more often (56% of responses) used by the participants, although only the mean score of the item covering social influence by colleagues was higher than 3.0. The mean score of the item covering unforeseen things was the only one in the direction of the disagree point along the continuum (mean score < 2.0). Participants also responded, on average, more frequently with *partly agree* or *agree* to the items in the motivation scale (70% of responses). Ten out of 18 items had a mean score higher than 3.0. Three out of 18 had a mean score lower than 2.0.

**Table 2:** Summary of mean (SD) item scores and frequency scores of the response options (*n* = 231–247†)

Sub‐scale and items	Mean (SD) score‡	Response options (*n*)				
		Disagree	Partly disagree	Neutral	Partly agree	Agree
Capability						
I have the practical *skills* to physically activate the people I support.	3.26 (0.8)	0	9	26	92	105
I am able to physically activate the people I support no matter how many *other tasks* I have.	2.08 (1.3)	28	61	35	78	29
I know how to physically activate the people I support when they are struggling with their *motivation*.	2.81 (0.9)	5	20	34	129	44
I do not forget to physically activate the people I support on a *daily basis*.	2.28 (1.4)	31	45	38	65	53
I *know* appropriate physical activities for the people I support.	3.09 (0.9)	2	19	19	109	83
I know how to physically activate the people I support even when I have *little time*.	2.46 (1.2)	17	35	45	92	42
I know *why* physical activity is *good* for the people I support.	3.84 (0.4)	0	1	4	27	200
Opportunity						
It is the *culture* within my organisation that people with ID are physically activated.	2.77 (1.1)	11	23	44	103	66
At my work location, I do not regularly have to deal with *unforeseen things* that result in failing to physically activate.	1.35 (1.2)	73	85	30	48	11
I offer physical activities regardless of *weather* conditions.	2.50 (1.2)	19	40	45	84	59
At my work location, there are *materials* available to carry out physical activities.	2.79 (1.3)	21	32	21	78	95
The organisation in which I work has a *budget* available for physical activities for the people I support.	2.26 (1.3)	31	32	72	62	48
I can count on the *support of family* if I want to physically activate their relatives more.	2.33 (1.3)	23	42	64	64	53
I offer physical activities regardless of the availability of *transport*.	2.47 (1.3)	27	30	55	68	65
At my work location, there is *time scheduled* for carrying out physical activities.	2.33 (1.5)	43	35	34	65	68
Within our *team*, it is self‐evident that the people we support will be physically activated.	2.90 (1.1)	9	23	34	97	82
The *organisation* in which I work offers me *time* to physically activate the people I support.	2.28 (1.3)	31	49	37	74	52
The *organisation* in which I work has a clear *policy* concerning physical activity.	2.42 (1.2)	22	31	66	75	51
I can count on the support of my *colleagues* if I want to physically activate the people we support more.	3.17 (0.9)	2	11	32	98	102
*Family expects* me and my colleagues to physically activate their relatives.	2.72 (1.1)	13	21	51	90	64
I offer physical activity regardless of the *accessibility* of the *environment*.	2.38 (1.2)	16	41	63	75	44
I can count on *support* from my *organisation* if I want to physically activate the people I support more.	2.59 (1.0)	7	30	66	88	49
I am not dependent on *volunteers* for offering physical activities to the people I support.	2.14 (1.4)	36	63	28	54	57
Motivation						
I physically activate the people I support, because I expect them to *improve* their *contact* with others.	2.64 (1.1)	16	24	43	115	49
Physically activating the people I support is *easy to maintain*.	2.19 (1.2)	23	59	38	103	24
I physically activate the people I support more often after I have *experienced success* (I was successful the last time).	2.89 (1.1)	12	19	32	105	79
I pay attention to physical activity no matter whether or not a *problem* for the people I support will be *solved*.	3.05 (1.0)	6	17	21	118	85
It is not very likely that I will give higher *priority* to something other than physical activity.	1.52 (1.1)	52	77	68	38	12
I play an *important role* in stimulating physical activities among the people I support.	3.30 (0.9)	3	12	27	71	134
In my work I am *happy* about physically activating the people I support.	3.49 (0.7)	1	5	14	80	147
If I see that physical activity has a *positive effect* on the people I support, then I physically activate them more.	3.57 (0.6)	0	2	10	79	156
I physically activate the people I support, because I expect it to be good for their *health*.	3.60 (0.6)	0	2	12	69	164
I find it *easy to* physically *activate* the people I support.	2.13 (1.2)	26	59	47	87	28
I experience *no stress* in my work by having to physically activate the people I support.	2.49 (1.3)	15	53	43	69	67
I think physically activating the people I support is a *nice part* of my work.	3.31 (0.8)	0	6	31	90	120
I physically activate the people I support more when I *get something in return* (e.g. better contact with them).	3.40 (0.8)	2	10	13	83	139
I pay attention to physical activity no matter whether or not a *goal* has been set up for a person I support.	3.22 (0.9)	6	9	22	98	112
I am *not worried* about the things that can go wrong when physically activating the people I support.	1.99 (1.3)	35	66	41	77	28
As a direct support professional, I am *responsible* for physically activating the people I support.	3.37 (0.8)	2	10	14	89	132
I physically activate the people I support, because I expect them to *develop better* as a result.	3.11 (0.9)	5	11	30	106	95
I experience *no problems* when carrying out physical activities with the people I support.	1.73 (1.3)	44	82	42	55	24
Total		715	1301	1491	3347	3117

† Available responses for the items ranged from 231 to 247.

‡ Item scores could vary from 0 (*disagree*) to 4 (*agree*), with a higher score indicating a greater degree of being facilitative.

ID, intellectual disability.

### Psychometric properties of the capability scale

The calibrated graded response model for the capability scale explained 50% of the data variance. Factor loadings ranged from 0.56 to 0.82. The estimated slope parameters for the items in the capability scale range from 1.14 to 2.41 (Table 3) and confirm that estimating a unique *α* for each item was reasonable. This also indicates that all the items have a satisfactory distinction power. The category threshold parameters range from −2.08 to 1.99. Within each item, the distance between the lowest and highest category threshold parameters is 1.74 to 4.07 units, which means that the capability construct is well covered. In addition, as shown in Table 3, the standard errors for the estimated IRT parameters indicate that they are estimated with good precision. The estimated IRT scores for the participants range from −2.57 to 1.99, which are not on the same metric as the category thresholds. Two participants have estimates IRT scores lower than −2.08.

**Table 3:** Item response theory parameters for the graded response models†

Sub‐scale and items	*α* (SE)	*β*_1_ (SE)	*β*_2_ (SE)	*β*_3_ (SE)	*β*_4_ (SE)
Capability					
Skills	1.47 (0.24)	−1.58 (0.27)	0.16 (0.18)	—	—
Other tasks	1.19 (0.21)	−2.08 (0.28)	−0.57 (0.18)	0.09 (0.17)	1.99 (0.24)
Motivation	2.15 (0.29)	−1.59 (0.37)	−0.87 (0.28)	1.13 (0.31)	—
Daily basis	1.68 (0.25)	−1.58 (0.30)	−0.66 (0.21)	−0.07 (0.19)	1.05 (0.24)
Knowledge	2.37 (0.40)	−1.63 (0.48)	−1.14 (0.39)	0.43 (0.26)	—
Little time	2.41 (0.35)	−1.79 (0.52)	−0.97 (0.34)	−0.30 (0.25)	1.12 (0.37)
Why good	1.14 (0.26)	−1.96	—	—	—
Opportunity					
Culture	0.82 (0.18)	−2.50 (0.21)	−1.06 (0.16)	1.39 (0.17)	—
Unforeseen things	0.85 (0.17)	−1.19 (0.16)	0.72 (0.15)	1.51 (0.16)	3.99 (0.32)
Weather	1.32 (0.21)	−2.39 (0.31)	−1.18 (0.21)	−0.35 (0.17)	1.15 (0.20)
Materials	0.98 (0.19)	−2.76 (0.25)	−1.53 (0.18)	−1.01 (0.17)	0.56 (0.16)
Budget	0.98 (0.18)	−2.32 (0.24)	−1.29 (0.17)	0.26 (0.15)	1.69 (0.19)
Family support	0.88 (0.17)	−2.89 (0.23)	−1.26 (0.16)	0.16 (0.15)	1.67 (0.18)
Transport	0.80 (0.18)	−2.91 (0.22)	−1.65 (0.17)	−0.21 (0.15)	1.44 (0.16)
Time scheduled	1.69 (0.24)	−1.34 (0.26)	−0.70 (0.21)	−0.21 (0.20)	0.80 (0.20)
Team	1.94 (0.26)	−1.50 (0.32)	−0.82 (0.25)	0.56 (0.22)	—
Organisation time	1.92 (0.26)	−1.54 (0.32)	−0.62 (0.23)	−0.10 (0.20)	1.05 (0.25)
Organisation policy	1.57 (0.24)	−2.00 (0.30)	−1.16 (0.23)	−0.07 (0.18)	1.20 (0.23)
Collegiate support	1.25 (0.23)	−2.76 (0.33)	−1.46 (0.21)	0.37 (0.17)	—
Family expectations	0.77 (0.17)	−4.01 (0.30)	−2.56 (0.20)	−0.86 (0.15)	1.46 (0.17)
Accessibility environment	0.93 (0.18)	−3.19 (0.28)	−1.46 (0.18)	−0.02 (0.15)	1.84 (0.19)
Organisational support	1.12 (0.19)	−1.85 (0.22)	−0.31 (0.16)	1.51 (0.20)	—
Volunteers	0.84 (0.18)	−2.30 (0.21)	−0.46 (0.15)	0.19 (0.15)	1.58 (0.18)
Motivation					
Improve contact	0.70 (0.15)	−4.10 (0.28)	−2.60 (0.19)	−1.13 (0.15)	2.16 (0.17)
Easy to maintain	1.51 (0.26)	−2.01 (0.32)	−0.71 (0.21)	−0.12 (0.18)	2.00 (0.31)
Success experiences	0.41 (0.14)	−7.35 (0.31)	−4.91 (0.20)	−2.76 (0.16)	1.85 (0.14)
Solve problems	1.10 (0.18)	−2.48 (0.27)	−1.71 (0.21)	0.72 (0.17)	—
Priority	1.04 (0.18)	−1.56 (0.21)	0.01 (0.15)	1.53 (0.18)	3.30 (0.33)
Important role	1.03 (0.21)	−3.07 (0.31)	−1.83 (0.21)	−0.22 (0.16)	—
Happiness	1.24 (0.22)	−2.38 (0.28)	−0.40 (0.17)	—	—
Positive effect	1.04 (0.22)	−3.28 (0.34)	−0.64 (0.16)	—	—
Health effects	1.17 (0.30)	−2.84 (0.33)	−0.73 (0.18)	—	—
Easy to activate	1.75 (0.29)	−1.75 (0.34)	−0.60 (0.22)	0.08 (0.19)	1.73 (0.34)
No stress	1.45 (0.22)	−2.42 (0.34)	−0.95 (0.21)	−0.25 (0.18)	0.91 (0.20)
Nice part	2.30 (0.40)	−1.28 (0.39)	0.01 (0.23)	—	—
Get something in return	1.08 (0.26)	−3.21 (0.36)	−2.39 (0.26)	−0.29 (0.16)	—
Goal settings	1.47 (0.27)	−2.40 (0.34)	−1.55 (0.25)	0.17 (0.18)	—
Worriless	0.43 (0.15)	−4.38 (0.19)	−0.95 (0.14)	0.69 (0.13)	4.97 (0.21)
Responsibility	1.19 (0.21)	−2.97 (0.35)	−2.20 (0.27)	−0.17 (0.17)	—
Better development	1.07 (0.21)	−2.88 (0.30)	−1.66 (0.21)	0.50 (0.16)	—
No problems	1.17 (0.23)	−1.62 (0.22)	0.01 (0.16)	0.80 (0.17)	2.35 (0.30)

† A reasonable range for *α* is from 0.5 to 3.0 (Baker, as cited in Toland 2014) and for *β* from −3 to 3 (Toland 2014).

**Figure 1:**
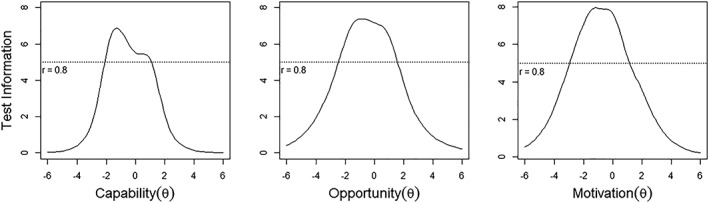
Test information function per sub‐scale.

In Fig. 1, the test information function for the capability scale demonstrates that most of the test information is below the middle ranges of the capability construct and that the precision of the capability scale peaked near −1.2. The IIFs for the capability items are provided in the Appendix. Direct support professionals in the capability construct between −2.2 and 1.2 are likely to be measured with the greatest reliability (>0.8; see also Fig. 1). Marginal reliability for the capability scale is 0.84.

### Psychometric properties of the opportunity scale

The calibrated graded response model for the opportunity scale explained a proportional variance of 0.31, where factor loadings ranged from 0.41 to 0.75. The estimated slope parameters for the items in the opportunity scale range from 0.77 to 1.94, which indicates that all the items have a satisfactory distinction power (Table 3). The category threshold parameters range from −4.01 to 3.99. Within each item, the distance between the lowest and highest category threshold parameters is 2.06 to 5.47 units. The opportunity scale covers the underlying construct well. The standard errors for the estimated IRT parameters are reasonably small (0.15 to 0.33) and indicate that the parameters were estimated with suitable precision. The estimated IRT scores for the 247 participants range from −2.73 to 2.21, which are on the same metric as the category thresholds.

The test information function indicates that most of the information is found around the middle ranges of the opportunity construct (Fig. 1). The IIF for the opportunity items is provided in the Appendix. Direct support professionals in the opportunity construct between −2.2 and 1.8 are likely to be measured with the greatest reliability (>0.8; Fig. 1). Marginal reliability for the opportunity scale is 0.87.

### Psychometric properties of the motivation scale

The calibrated graded response model for the motivation scale explained a proportional variance of 0.32. Factor loadings ranged from 0.23 to 0.80. The estimated slope parameters for the items in the motivation scale range from 0.43 to 2.30 (Table 3). The majority of the items of the motivation scale have a satisfactory distinction power. The category threshold parameters range from −7.35 to 4.95. Within each item, the distance between the lowest and highest category threshold parameters is 1.291 to 9.351 units, which means that the motivation construct is broadly covered. The parameters for the motivation scale are estimated with satisfactory precision, apart from a standard error of 0.40 for the slope parameter of affinity. The estimated IRT scores for the 247 participants range from −3.20 to 2.81, which are on the same metric as the category thresholds of the motivation scale.

The test information function, as shown in Fig. 1, indicates that there is more information below the middle ranges of the motivation construct. The IIF for the motivation items is provided in the Appendix. Direct support professionals on the motivation construct between −3.0 and 1.3 are likely to be measured with the greatest reliability (>0.8; Fig. 1). Marginal reliability for the motivation scale is 0.87.

## Discussion

The aim of this study was to develop and validate a tool to measure the behaviour of direct support professionals in terms of their physical‐activity support for people with ID. The development of the tool was theoretically well founded, and experts were involved to ensure its content validity. The study's main objective was to evaluate the psychometric properties of the tool to facilitate research in the field. With IRT models, we analysed the construct validity and reliability of the three theory‐driven behaviour scales for direct support professionals of people with ID. In addition, the IRT models allowed the performance of individual items to be evaluated.

The results demonstrate good construct validity for the capability and opportunity scales and reasonable construct validity for the motivation scale. In the motivation scale, two of the items relate less to the construct measured (i.e. slope parameters were unsatisfactory). These items, however, did not correlate with items from the capability and opportunity scales. Their retention in these scales is warranted as long as the IRT score estimates, which take into account item properties, are used. Furthermore, removing items is only allowed when it does not destroy content validity (Toland 2014). The results also prove that the capability, opportunity and motivation scales are reliable, with good measurement precision along the continua. Additionally, the ranges of the threshold parameters ensured that all of the scale levels were represented in the current scale items. The scales, in their current stage, can distinguish satisfactorily between direct support professionals over the entire range of capability, opportunity and motivation levels.

This study is not without limitations. Content experts were involved in the development of the different sub‐scales. Content experts' feedback can be subjective; thus, the study might be subjected to bias that may exist between these two experts. However, the potential participants were also asked to suggest other items for the tool, which helped minimise this limitation. Additionally, a number of potential participants (*n* = 38) exited the online tool before completion, and this study's design did not allow for the reasons for quitting to be identified. It might be that these direct support professionals did not agree with the content of the tool. In future, we should incorporate the rationale behind the reason for not completing. The same applies to percentage of missing data for some items (range: 0 to 6.4). However, it can be assumed that these limitations did not significantly affect the results presented in the current study. In IRT models, because of the invariance property, a non‐random sample from the population of interest can be used (De Mars 2010). Furthermore, IRT models are perfect for handling data with missing values.

Based on the results found, the tool is potentially useful in assessing direct support professional behaviour vis‐à‐vis their support of physical activity; this study's data can already be used to identify areas and target groups for future interventions and policies. Additionally, based on this study's data, we can recommend minor changes to the scales before being used in practice, along with further psychometric research.

The content in terms of the difficulty of some of the items could be adjusted. For example, the category threshold estimates for the response options of *partly agree* and *agree* for the item ‘unforeseen things’ were extremely high. It is expected that only respondents who score very high on the opportunity continuum will answer this item positively. In contrast, the category threshold estimates for the item ‘family expectations’ were extremely low. Respondents with both low and high levels in terms of the opportunities afforded will respond neutrally or positively to this item. The same applies to a number of items in the motivation scale (e.g. success experiences or worriless). Changing the content in terms of difficulty of these items could also contribute to the scale's construct validity.

The scales in their current state are particularly reliable in determining those who score on the lower levels of capability, opportunity and motivation. To improve the distinctiveness and reliability of the scales, we recommend adding more items to the capability scale with thresholds category above 1.2, to the opportunity scale above 1.8 and to the motivation scale above 1.3. However, additional items are not necessary when the intention is to use the scales in the clinical field to principally identify those direct support professionals who can benefit from an intervention or change in policy.

Another recommendation for practical purposes may be to shorten the tool, especially for the opportunity and motivation scales. In reference to the study results, some items both reflect the same concept and have overlapping IIFs (Appendix). In the context of a critical look at content validity, one might consider removing one of the items or merging them. For example, although various aspects were addressed, there are multiple items covering the concept of organisation. Policymakers might choose to merge the items for organisational support, time provided and budget. Alternatively or in addition, policymakers might choose between the item on family expectations and the one on family support, because both function in a similar way in this study's data. However, the psychometric properties would then have to be re‐examined, which can be carried out in close collaboration with researchers.

Future psychometric research on the tool should incorporate participant‐centred research methods, such as interviews and behavioural observations. Interviews that investigate the perspectives of direct support professionals for different positions on the continua or with striking combinations will contribute to validation of the tool. Accordingly, this can help to improve our understanding of direct support professional behaviour. Behavioural observations allow researchers to measure the tool's correlation with the actual physical‐activity support for people with ID. In addition, future research should assess the tool's intra‐rater reliability and its sensitivity to change over time. This will enable the use of this tool to monitor and evaluate intervention functions and organisational policy change focused on improving the physical‐activity support.

## Conclusions

This study focused on the development of a tool to measure the behaviour of direct support professionals and has provided evidence on preliminary content, construct and reliability. The tool can be used to measure the capability, motivations and opportunities afforded to carry out physical‐activity support among direct support professionals who support people with ID. The tool can also be used to measure differences between direct support professionals in terms of their own characteristics, the diversity of the people with whom they work and their environmental context. Moreover, this study's results have addressed theoretical support for the model of direct support professional behaviour in the physical‐activity support for people with ID.

## Source of funding

This research did not receive any specific grants from funding agencies in the public, commercial or not for‐profit sectors.

## Conflict of Interest

There was no conflict of interest, and no restrictions were imposed on the publication of results.

## Figures and Tables

**Figure A.1:**
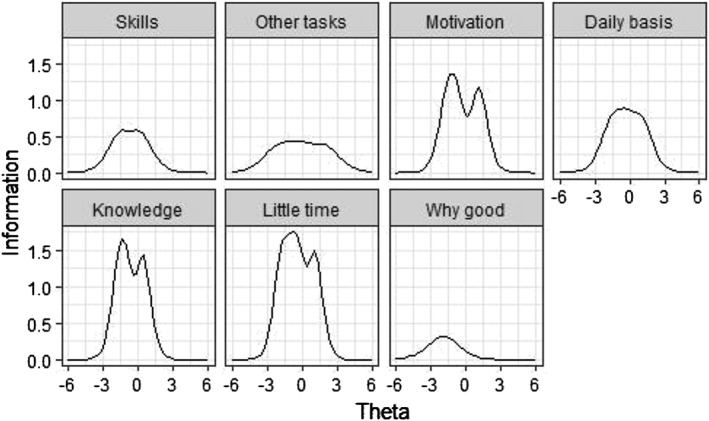
Item information function per item in the capability scale.

**Figure A.2:**
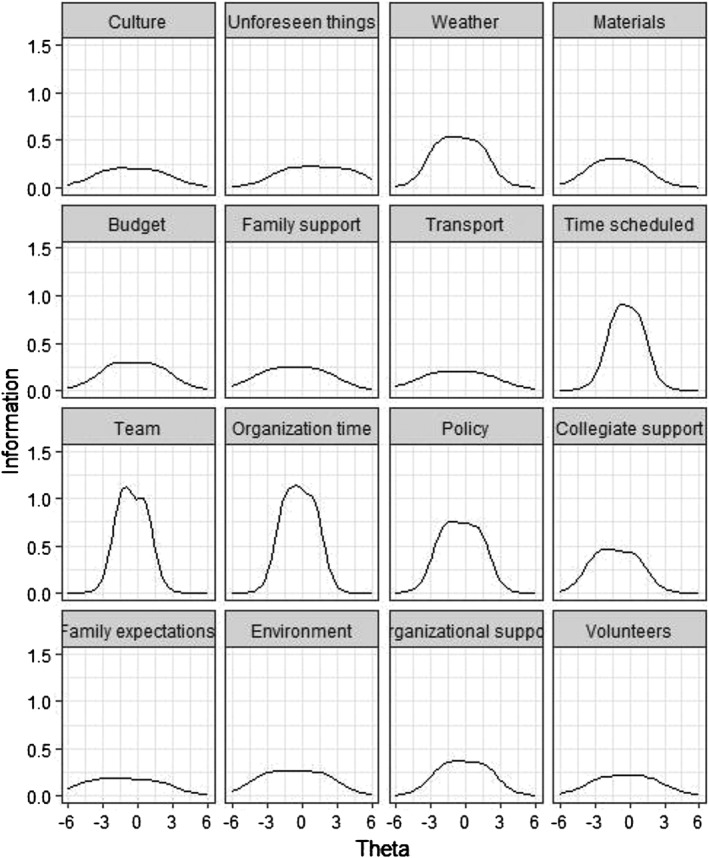
Item information function per item in the opportunity scale.

**Figure A.3:**
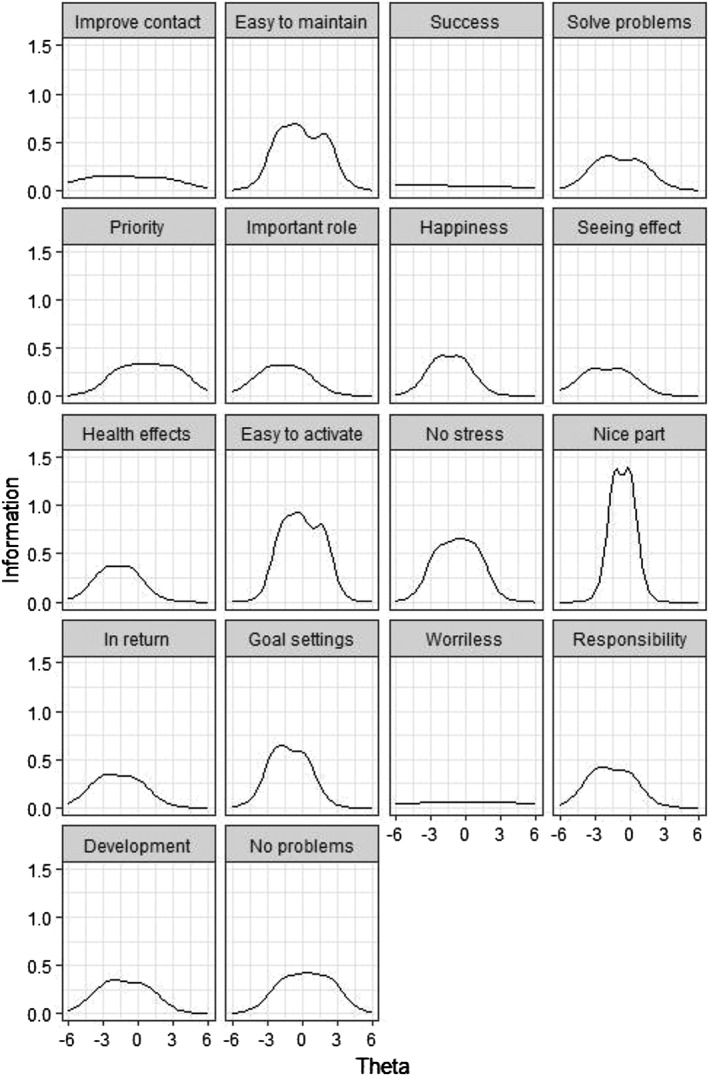
Item information function per item in the motivation scale.
